# The validated French CFAbd‐Score reveals a lower burden of gastrointestinal symptoms in patients on Elexacaftor/Tezacaftor/Ivacaftor

**DOI:** 10.1002/jpn3.70244

**Published:** 2025-11-03

**Authors:** Isabelle Sermet‐Gaudelus, Pauline Sadrieh, Anne Sophie Bonnel, Raphael Enaud, Nathalie Wizla, Jeanne Languepin, Ambre Nangui, Audrey Deelawon, Johanna Rave, Samira Dabelow, Anton Barucha, Carlos Zagoya, Franziska Duckstein, Jochen Georg Mainz

**Affiliations:** ^1^ Université de Paris Paris France; ^2^ INSERM U1151, Institut Necker Enfants Malades Paris France; ^3^ Centre de Référence Maladie Rare‐Mucoviscidose Hôpital Necker Enfants Malades Paris France; ^4^ European Reference Network‐Lung Frankfurt Germany; ^5^ Cystic Fibrosis Centre Brandenburg Medical School (MHB) University, Klinikum Westbrandenburg Brandenburg an der Havel Germany; ^6^ Faculty of Health Sciences Joint Faculty of the Brandenburg University of Technology Cottbus‐Senftenberg, the Brandenburg Medical School Theodor Fontane and the University of Potsdam Potsdam Germany; ^7^ Hôpital André Mignot Le Chesnay France; ^8^ Centre de Recherche Cardio‐Thoracique de Bordeaux Univ. Bordeaux Bordeaux France; ^9^ CRCM Pédiatrique Bordeaux France; ^10^ CRCM Pédiatrique Hôpital Jeanne de Flandre Lille France; ^11^ CRCM Pédiatrique Centre Hospitalier Universitaire Limoges France; ^12^ Department of Gastroenterology Brandenburg Medical School (Theodor Fontane) Brandenburg an der Havel Germany

**Keywords:** abdominal symptoms, CFTR, modulator therapy, patient‐reported outcome measure, scoring

## Abstract

**Objectives:**

Multiorgan abdominal involvement is a hallmark of Cystic fibrosis (CF). The CFAbd‐Score^©^ is the first CF‐specific gastrointestinal patient reported outcome‐measure (PROM) developed following FDA‐guidelines. The PROM has proved to sensitively differentiate people with CF (pwCF) from healthy controls (HC). Furthermore, higher Scores in pwCF are associated with pancreatic insufficiency and history of surgery. Recently, in two large cohorts of pwCF, the PROM revealed significant and clinically meaningful improvements in abdominal symptoms (AS) in pwCF treated with Elexacaftor‐Tezacaftor‐Ivacaftor (ETI). In the present publication, we aim to validate the translated, linguistically and cross‐culturally adapted French version of the CFAbd‐Score^©^.

**Methods:**

Validation was performed following two forward translations and a review process by a German professional medical translator. The reviewed consensus version was psychometrically evaluated for validity and reliability in French‐speaking pwCF and HC cohorts, as well as in pwCF with and without ETI.

**Results:**

A total of 64 French‐speaking pwCF (mean age: 12.5 years) and 58 HC were included. Median CFAbd‐Score^©^ in pwCF without ETI was significantly higher than in HC (18.2 vs*.* 8.7 points/*p* < 0.01), while Scores from ETI‐treated pwCF were almost as low as the HC‐range in many domains. Scores from ETI‐treated and nontreated pwCF differed significantly (8.4 vs*.* 18.2 points/*p* < 0.01).

**Conclusion:**

The French version of the CFAbd‐Score has proved to be reliable and sensitive to capture AS in French‐speaking pwCF and to distinguish pwCF from HC. Furthermore, consistent with previous findings, the PROM sensitively detects ETI‐related differences in AS of pwCF with and without ETI therapy, and thus can be used in research and clinical routine.

## Abstract (highlights)

**What is Known:**

- The CFAbd‐Score is the first Cystic fibrosis (CF)‐specific gastrointestinal patient‐reported outcome‐measure.
- It has been developed and validated according to recommendations by the Food and Drug Administration (FDA).

**What is New:**

- The CFAbd‐core is now made available to French‐speaking people with CF (pwCF), through translation and cultural adaptation as suggested by the International Society for Pharmacoeconomics and Outcomes Research and the FDA.
- The CFAbd‐Score in French reveals excellent psychometric properties, differentiating pwCF from healthy controls.
- CFAbd‐Scores result to be much higher in pwCF without Elexacaftor‐Tezacaftor‐Ivacaftor therapy, which accords well to improvements found in our previous prospective studies.

## Graphical abstract

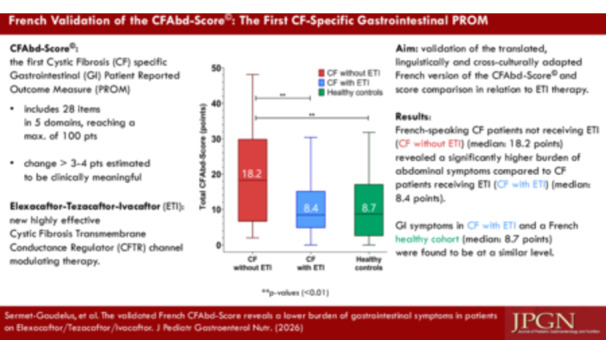

## 1 INTRODUCTION

Cystic fibrosis (CF) is the most frequent inherited disease in people of European descent. Caused by a mutation in the CF transmembrane conductance regulator (CFTR) gene, it results in defective or insufficient CFTR channels on epithelial surfaces of exocrine glands. This impairs the outflow of chloride, bicarbonate, and consequently water, producing highly viscous mucus in multiple organs.^1^ Symptoms occur in all organ systems where the CFTR channel is present, with disorders of the respiratory and digestive tracts being the most clinically relevant.^2^ Accordingly, abdominal symptoms (AS) are a hallmark of CF, starting already at birth in 10%–15% of patients with meconium ileus, regularly associated with exocrine pancreatic insufficiency, and later with gastroesophageal reflux disease (GERD), distal intestinal obstruction syndrome (DIOS), hepatobiliary involvement, and endocrine pancreatic insufficiency (diabetes).^3^ AS are consequently a major cause of alteration in the quality of life (QoL) of people with CF (pwCF).^4^

With the increasing life expectancy of pwCF, the need to understand AS is nowadays well‐recognized in CF communities, and relieving AS even ranked among the top 10 research priorities in CF in 2018 and subsequently in 2023.^5^, ^6^ However, until recently, there was a lack of a CF‐specific tool to measure and quantify AS in both clinical and research setups.^7^ To respond to this unmet need, Mainz et al. developed and validated the CFAbd‐Score, the first disease‐specific patient‐reported outcome measure (PROM) developed and validated to assess AS in pwCF, following guidelines of the Food and Drug Administration (FDA).^8^

In France, there are more than 7700 pwCF, of whom about 85% are already treated with Elexacaftor‐Tezacaftor‐Ivacaftor (ETI).^9^ Consequently, there was the need to validate this new effective tool for its use in French‐speaking pwCF.

We hypothesize that a validated French version of the CFAbd‐Score will provide the French‐speaking CF community, including pwCF, their proxies and CF specialists, with a standardized sensitive CF‐specific tool to capture GI symptoms. Accordingly, the aim of the present publication is to outline the translation, cultural adaptation, and validation of the CFAbd‐Score in French, including the consultation of French CF specialists of different professions, in order to make the CF and GI‐specific tool available to French‐speaking pwCF. A secondary aim of this study was to use the new French version of the PROM to assess ETI‐related effects on AS from pwCF receiving the highly effective modulator therapy (HEMT), compared to nontreated patients and healthy controls (HCs).

## 2 METHODS

### 2.1 Ethics statement

The Research Ethics Committee of the Centre‐Université de Paris, IDF2, approved the study protocol, including provided written informed consent before data collection (AFSSAPS [ANSM] B1005423‐40, n° Eudract 2010‐A00392‐37; CPP IDF2: 2010‐05‐03‐3).

### 2.2 Study design

Before validation, the translation process was carried out considering the guidelines outlined by the International Society for Pharmacoeconomics and Outcomes Research (ISPOR) and the European Group for Health Management and QoL Assessment for translation and cultural adaptation.^10^, ^11^, ^12^ As part of the validation process, we analyzed the psychometric properties of the French version of the CFAbd‐Score according to FDA guidelines.

A first translation of the CFAbd‐Score to French was performed in 2018 and it was re‐translated in November 2019. The French version of the PROM for the present study was finalized by July 2021 following an interactive approach between the French and a German professional translator.

Pediatric and adult pwCF diagnosed by elevated sweat test above 60 mmol/L and or carrying two CF‐causing *CFTR* mutations were enrolled in the CF Center of the Centre‐Université de Paris, Hôpital Necker Enfants Malades for children and adults. Recruiting of patients and HCs took part between July 2021 and December 2022.

Exclusion criteria for all participants were acute exacerbations/infections and chronic gastrointestinal (GI) diseases, such as celiac disease and inflammatory bowel diseases, as well as incomplete questionnaires (more than 50% of missing answers). Furthermore, demographic and medical information from pwCF was collected, including *CFTR* genotype, medication use, and lung function.

The pwCF cohort comprised patients with and without treatment with the novel highly effective CFTR‐modulating therapy ETI. A HC cohort was also included, which consisted of parents and siblings of the enrolled pwCF and HC without history of CF in their family. All participants were fluent in French.

### 2.3 The CFAbd‐Score

Originally developed in German, the CFAbd‐Score, the first CF‐specific PROM, which was aimed to be translated into French, comprises 28 items attributed to five domains.^8^ It was developed and validated according to FDA and COSMIN guidelines including pwCF and their proxies in cognitive interviews and with input from CF specialists from various disciplines.^13^, ^14^, ^15^ The five domains: pain symptoms (four items), disorders of bowel movement (eight items), disorders of eating and appetite (five items), GERD symptoms (three items), and impairment of quality of life (QoL, eight items), as well as the total CFAbd‐Score are calculated using a Score‐specific algorithm developed following FDA guidelines resulting in a maximum of 100 points with higher Scores representing a higher burden of symptoms. Validation of the initial German version of the CFAbd‐Score revealed excellent internal consistency and known‐group validity.^8^ Subsequently, the PROM was proved to detect changes in two international studies, prospectively following up 107 and 108 pwCF from Germany, the United Kingdom, and Ireland, showing a significant and clinically meaningful decrease in CFAbd‐Score levels after initiation of the new triple modulator therapy with ETI.^16^, ^17^

### 2.4 Descriptive analysis French translation

**Figure 1:**
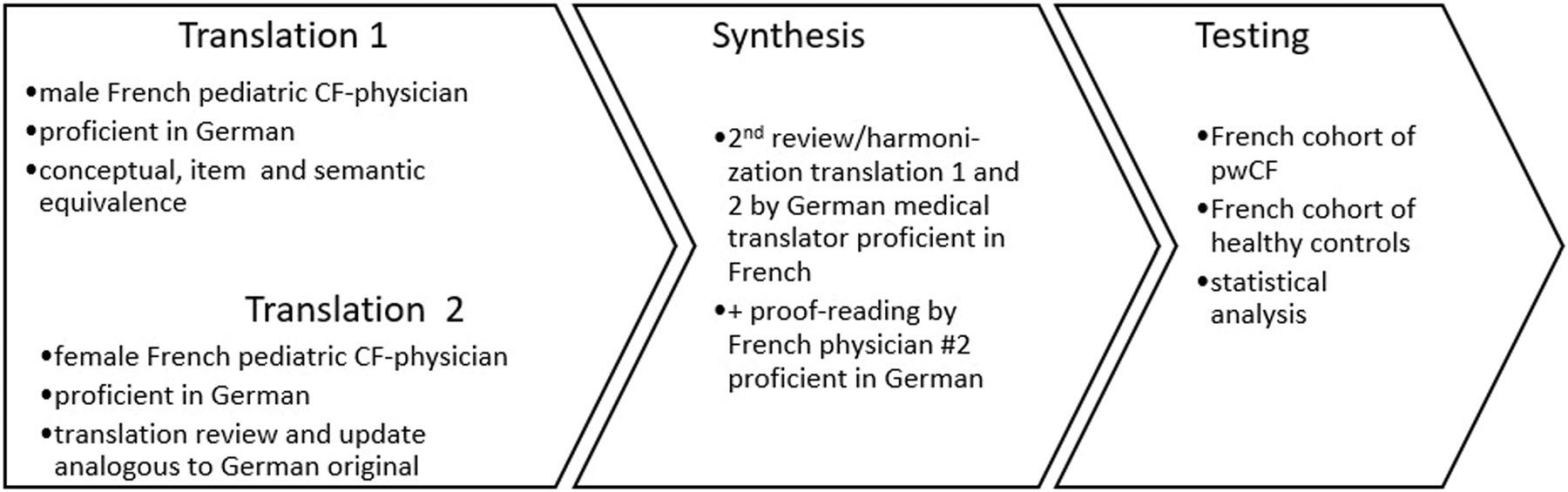
Translation and cultural adaptation process of the German CFAbd‐Score. CF, Cystic fibrosis; pwCF, people with CF.

Following ISPOR guidelines, two independent pediatric CF physicians, who are native French speakers and fluent in German, carried out forward source‐oriented translations of the most recent German version of the CFAbd‐Score into a French target text. The involvement of pediatric CF physicians familiar with the Score ensured preserving the semantic meaning implicit in the items. A German professional medical translator proficient in French proofread, re‐checked, and culturally adapted both forward translations into a synthesized target culture‐oriented version. The French and German medical translators reached a consensus on the final French version and finalized formatting, typesettin,g and proofreading before testing the final consensus version for its validity and reliability. The process is illustrated in Figure 1. All items included in the original German version were retained in the final French version of the CFAbd‐Score as they were deemed relevant by a CF expert group consisting of CF physicians and CF caretakers.

### 2.5 Statistical analysis

Normality assumptions in the data distributions were verified using the Shapiro‐Wilk test. Differences in the total CFAbd‐Score and its five domains between the three groups, that is, pwCF receiving ETI therapy, pwCF not receiving ETI and the HC group, were conducted with the Kruskal–Wallis test. Pairwise comparisons using Mann–Whitney *U* tests were conducted when the *p*‐value resulting from the Kruskal–Wallis test was <0.05.

All statistical analyses were performed using R version 3.6.3.

### 2.6 Psychometric evaluation

For psychometric evaluation of the French version of the CFAbd‐Score, two cohorts were considered. One cohort consisted of pwCF who met the diagnostic criteria of CF and were native French speakers or fluent in French. The second cohort was the HC group.

## 3 RESULTS

### 3.1 Characteristics of study sample

A total of 64 pwCF (28 females) recruited at the CF‐Center at the Institute Necker Enfant Malades were included in the analysis. Median age was 12.5 years (interquartile range [IQR]: 7.8–15.3, min: 3 years, max: 41 years). The HC cohort consisted of 58 people (45 females and 13 males) with a median age of 37.5 years (IQR: 32.3–44.8; min: 3 years, max: 57 years). Patients aged ≥12 years completed the questionnaires by themselves. Younger children, however, were supervised by their parents/guardians (by‐proxy implementation).

Table 1 shows the demographics and clinical characteristics of the pwCF included in the study.

**Table 1:** Demographics and clinical characteristics of pwCF included in the study.

		All pwCF	PwCF without ETI	PwCF with ETI	Healthy controls
		*n* = 64	*n* = 31	*n* = 33	*n* = 58
		*n* (%)			
Sex	Female	28 (44)	14 (45)	14 (42)	45 (78)
	Male	36 (56)	17 (55)	19 (58)	13 (22)
Age	<6 years	10 (16)	10 (32)	0 (0)	1 (2)
	6–<12 years	21 (32)	11 (36)	10 (30)	1 (2)
	≥12 years	33 (52)	10 (32)	23 (70)	56 (96)
Genotype	F508del homozygous	28 (44)	10 (32)	18 (55)	
	F508del heterozygous w/o G551D	12 (19)	1 (3)	11 (33)	
	F508del heterozygous with G551D	0 (0)	0 (0)	0 (0)	
	Other	24 (37)	20 (65)	4 (12)	
CFTR‐modulating therapy	ETI	33 (52)	0 (0)	33 (100)	
	Lumacaftor/ivacaftor	8 (12)	8 (26)	0 (0)	
	None	23 (36)	23 (74)	0 (0)	

Abbreviations: CF, Cystic fibrosis; CFTR, CF transmembrane conductance regulator; ETI, Elexacaftor‐Tezacaftor‐Ivacaftor; pwCF, people with CF.

### 3.2 Known‐groups validity

**Figure 2:**
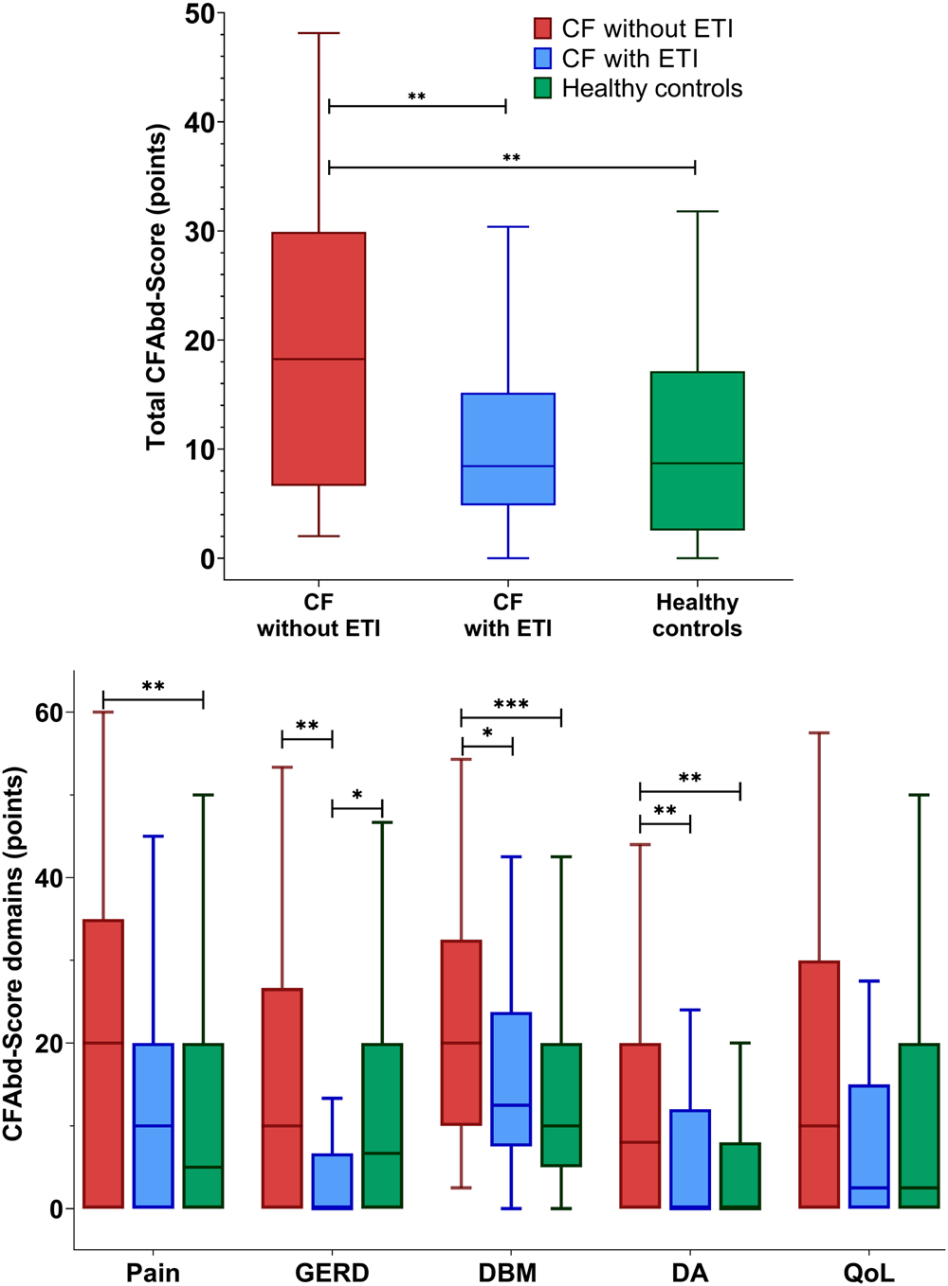
Total CFAbd‐Score (top) and its five domains (bottom), that is, pain, GERD, disorders of bowel movements, disorders of appetite and QoL, in the three different subgroups of French pwCF included in this study. *p*‐Values are represented as: *p < 0.05, **p < 0.01, and ***p < 0.001. CF, Cystic fibrosis; DA, disorders of appetite; DBM, disorders of bowel movement; ETI, Elexacaftor‐Tezacaftor‐Ivacaftor; GERD, gastroesophageal reflux; pwCF, people with CF; QoL, quality of life.

Known‐groups validity was assessed by comparing scores among the two subgroups of pwCF, that is, pwCF on ETI therapy (*n* = 33) and pwCF without ETI therapy (*n* = 31), and the HC group (*n* = 58). The total CFAbd‐Score resulted highest in pwCF without ETI therapy with a median total CFAbd‐Score of 18.2 points, being significantly (*p* < 0.01) and markedly higher (53%) than the median of 8.4 points in pwCF receiving ETI therapy. Total CFAbd‐Scores also resulted significantly (*p* < 0.01) higher (52%) in pwCF without ETI therapy, compared to HC (Figure 2).

Regarding the five domains of the PROM, median scores in pwCF without ETI therapy were also significantly higher than in HC for the domains “pain” (median: 20 points vs*.* 5 points), “disorders of bowel movement” (median: 20 points vs*.* 10 points,) and “disorders of appetite” (median: 8 points vs*.* 0 points). Concomitantly, median scores from pwCF without ETI therapy were significantly (*p* < 0.05) higher than median scores from pwCF on ETI therapy for the domains “GERD” (median: 10 points vs*.* 0 points), “disorders of bowel movement” (median: 12.5 points vs*.* 10 points) and “disorders of appetite” (median: 8 points vs*.* 0 points), see Table 2.

**Table 2:** Total CFAbd‐Scores and its five domains, that is, pain, GERD, DBM, DA, and GI‐related impairment of QoL, compared among pwCF with and without ETI‐therapy and HCs.

		pwCF without ETI (median [IQR]) *N* = 31	pwCF with ETI (median [IQR]) *N* = 33	HCs (median [IQR]) *N* = 72	*p*‐values
Total CFAbd‐Score		18.2 (7.5–29.2)	8.4 (5.1–14.4)	8.7 (2.8–16.7)	*p*_1_ < 0.01
					*p*_2_ < 0.01
Domains	Pain	20 (2.5–35)	10 (0–20)	5.0 (0–20)	*p*_1_ < 0.01
	GERD	10 (0–23.3)	0 (0–6.7)	6.7 (0–20)	*p*_2_ < 0.01
					*p*_3_ < 0.05
	DBM	20 (12.5–32.5)	12.5 (7.5–22.5)	10 (5–20)	*p*_1_ < 0.001
					*p*_2_ < 0.05
	DA	8 (0–20)	0 (0–12)	0 (0–8)	*p*_1_ < 0.01
					*p*_2_ < 0.01
	QoL	10 (0–27.5)	2.5 (0–15)	2.5 (0–17.5)	*p*_1_ < 0.05

*Note*: *p*_1_: *p*‐value for the comparisons between pwCF without ETI and HCs. *p*_2_: *p*‐value for the comparisons between pwCF with and without ETI. *p*_3_: *p*‐value for the comparisons between pwCF with ETI and the HC group.

Abbreviations: CF, Cystic fibrosis; DA, disorders of appetite; DBM, disorders of bowel movement; ETI, Elexacaftor‐Tezacaftor‐Ivacaftor; GERD, gastroesophageal reflux disease; GI, gastrointestinal; HC, healthy control; IQR, interquartile range; pwCF, people with CF; QoL, quality of life.

Differences between median scores from pwCF on ETI and the HC group attained significance only for the domain GERD (median, IQR for HC group: 6.7, 0–20; *p* < 0.05). Although median scores for the domain “QoL impairment” in the subgroup of pwCF without ETI therapy was highest among the three subgroups, these differences were not significant (Table 2). Comparisons including only pwCF and HC aged ≥12 years did not show significant differences, as a result of the relatively smaller group sizes of the pwCF subgroups (Table S1). Analogously, as a likely result of the small subgroup sizes, comparison of the total scores among the 4 age subgroups of pwCF, that is, <12 years with and without ETI (*n* = 10 and *n* = 21, respectively), as well as ≥12 years with and without ETI (*n* = 23 and *n* = 10, respectively) revealed no significant differences, except between the subgroups ETI and ≥12 years and no ETI and <12 years for both the total CFAbd‐Score and the disorders of appetite domain (Table S2).

## 4 DISCUSSION

With the increasingly longer life expectancy of pwCF, AS are increasingly coming to the clinical and scientific focus of CF care. In France, increasing life expectancy is now evident in the French CF registry, where in 2022, 62.5% of the registered 7743 pwCF were adults, in contrast to 17.8% in 1992.^9^ Furthermore, according to the French CF registry, in 2022, 80.2% of the pwCF included in the registry had abnormal exocrine pancreatic function, 27.4% were treated for GERD and 18.4% revealed CF‐related liver disease.^9^ In a French study by Sermet‐Gaudelus et al., the abdomen was identified as the most common location for pain in pediatric pwCF and the second most common location for pain in adult pwCF.^18^ Additionally, a French study found that elevated fecal calprotectin is associated with a higher burden of AS in children with CF, relevantly impairing their QoL.^19^

In this study, we successfully conducted a systematic translation, cultural adaptation, and psychometric validation of the CFAbd‐Score in French. This questionnaire is the first CF‐specific PROM aimed at assessing AS.^2^, ^8^, ^20^

Prompted by the lack of a CF‐specific validated GI PROM, the CFAbd‐Score was developed since 2015, with the aim to sensitively capture the pattern of GI symptoms caused by the specific pattern of organ involvement. Anticipating the introduction of highly effective CFTR modulators, a most relevant focus for the PROM was the ability to sensitively capture changes achieved by such effective therapeutic interventions.

Consequently, the CFAbd‐Score has proved to be sensitive to detect significant and marked changes before and after the start of ETI therapy in different cohorts of pwCF from Germany and the United Kingdom,^16^ and in the real world prospective observational RECOVER study, following longitudinal effects of ETI in pwCF from Ireland and the United Kingdom.^17^ In RECOVER total CFAbd‐Scores declined for −41% (−35%) after 2 months (12 months, respectively) of therapy with ETI (all *p* < 0.05). Scores in all of the five domains also resulted to decline significantly and to a clinically very relevant level: Pain −54% (41%), GERD −43 (31%), disorders of bowel movements −18% (−27%), disorders of appetite −61% (−34%) and GI‐related QoL −58% (−44%).

In agreement with that, in the present publication, we observed a difference of 9.8 points between pwCF with and without ETI treatment, that is, 53% higher in pwCF not receiving ETI (*p* < 0.01).

Such a difference, as well as differences reported in previous prospective studies assessing AS with the CFAbd‐Score before and during ETI treatment ^16^, ^17^ are greater than the previous estimation of the minimal clinically important difference accounting for 3−4 points for the total CFAbd‐Score.^21^

The present results, as well as previous ones, are in stark contrast to other studies performed with other not CF‐specific GI‐PROMs. With about 270 pwCF receiving a new therapy with ETI, these studies have implemented the PAC‐SYM, PAGI‐SYM, and PAC‐QoL questionnaires together with a modified Bristol Stool Scale, all of which have not been developed for the specific pattern of organ involvement in pwCF. Consequently, although some AS have been reported to significantly change in these studies, such changes have been deemed not clinically relevant by the studies' authors.^22^

An important result further supporting the translation and validity of the new French version of the CFAbd‐Score is the markedly significant difference between pwCF not receiving ETI‐therapy and HCs observed in the present study. This correlates well to previous studies using the CFAbd‐Score, for example, in its German, English, and Portuguese versions. Overall, pwCF scored significantly higher than HCs. Specifically, the median for the total CFAbd‐Score in pwCF without ETI therapy was significantly higher by 52% compared to the HC group, confirming that the CFAbd‐Score is able to sensitively differentiate known groups. In addition, significantly higher scores were found in three domains in pwCF not receiving ETI compared to HC (see Figure 2/Table 2).

Furthermore, in this study, total CFAbd‐Scores of ETI‐treated pwCF and those of HCs seem to be at a comparable level, despite the differences observed in the domains of “pain,” “GERD,” and “disorders of appetite.” Moreover, although improvement in the domains of “pain” and “disorders of bowel movement” does not reach “normality,” the median for the total CFAbd‐Score in the present French‐speaking HC cohort (8.7 [IQR: 2.8–16.7] points) was numerically equal to the estimated marginal mean (8.7 ± 1.0 points) found in the German HC group included in our previous study.^8^, ^16^

However, the fact that 86% of HC were immediate relatives of the included pwCF and, accordingly for this autosomal recessive disease, with higher probability of carrying *CFTR* mutations, raises further questions. In larger studies, carriers of a single *CFTR* mutation have revealed elevated risks of typical symptoms related to the multiorganic disease, including constipation and occlusion.^23^ This has been specially established for chronic rhinosinusitis and, most relevantly here, for pancreatitis.^24^, ^25^ Therefore, the question that remains open is whether a French‐speaking HC cohort without a relevant proportion of individuals carrying a single CFTR mutation could have attained lower scores when using our PROM, compared to the present HC cohort. If such a hypothesized effect was relevant, differences between the HC group and pwCF not treated with ETI would have been even more pronounced than in the present cohorts.

The imbalanced gender proportions in the HC group may pose a further limitation, potentially acting as a confounder. More specifically, the higher proportion of females in the HC cohort may not be directly comparable with the slightly higher proportion of males in the cohort of pwCF with and without ETI.

Nevertheless, the results presented here add on to previously published evidence supporting known‐groups validity by sensitively differentiating pwCF from HCs.^8^, ^26^ Additionally, in previous studies the CFAbd‐Score revealed higher scores in pwCF with history of abdominal surgery, with pancreatic insufficiency,^26^ and pancreatic anomalies in abdominal ultrasound,^20^ as well as with CF‐related diabetes.^21^

At present, the CFAbd‐Score is used worldwide in more than 25 countries and is available in 12 languages including German, English, French, Spanish, Portuguese, Italian, Greek, Turkish, Danish, Dutch, and Flemish. It is being increasingly implemented worldwide in clinical studies assessing correlations with clinical and diagnostic findings,^20^, ^26^, ^27^ as well as effects of therapeutic interventions, including aspects of international validation of the PROM.^2^, ^16^, ^17^, ^28^, ^29^, ^30^, ^31^

Structured translations and cross‐cultural adaptations of a PROM are necessary procedures in order for a PROM to preserve its consistency and accuracy attained in the language in which it was originally developed, for which the ISPOR has provided a series of guidelines and recommendations.^11^, ^12^, ^14^

In our clinical routine, the CFAbd‐Score relevantly impacts clinical decision‐making in daily practice. In our outpatient clinic the one‐sided CFAbd‐Score is provided to every pwCF presenting in the waiting room as, where it is completed by patients and proxies. Together with doctors and nutritionists, the issues addressed in the completed form are reviewed and compared with those identified in previously completed PROMs, for example. in regard to frequency of abdominal pain and frequency of stools, this often brings up a straightforward discussion on adherence and/or optimization of therapies.

Furthermore, the CFAbd‐Score has been implemented in the German CF registry, as an online tool, fostering its' availability to all German pwCF.

Limitations of this study include the small sample size across the age range and ETI subgroups, which prevented further subgroup comparisons. Nevertheless, the present cohort of pwCF with heterogeneous ETI therapy status reveals highly significant and clinically meaningful differences in AS of pwCF with and without treatment with the highly effective CFTR modulator. On the one hand, this indirectly confirms ETI to be effective in reducing the previously elevated burden of AS in pwCF.^16^, ^17^, ^32^ On the other hand, it also confirms the high sensitivity of the recently validated French version of the CFAbd‐Score to capture changes induced by highly effective therapies, in agreement with previous studies assessing ETI‐related AS improvement with the CFAbd‐Score.

A further limitation may be posed by the different age distributions between the HC and the pwCF subgroups, as only 4% of the HC subgroup consisted of children aged <12 years, whereas 48% of the pwCF cohort were in this age range.

## 5 CONCLUSION

The translated and adapted French version of the CFAbd‐Score has proved to be reliable, sensitive to capture AS in French‐speaking pwCF and able to differentiate AS in pwCF and healthy individuals. The CFAbd‐Score is now validated in French for routine care and research, providing an easily accessible tool in different languages and in different cultural spaces.

## CONFLICT OF INTEREST STATEMENT

The authors declare no conflict of interest.

## Supporting information


**Table S1.** CFAbd‐Score and its five domains for ages ≥12 years in the three subgroups: pwCF without ETI, pwCF on ETI and HC.

**Table S2.** CFAbd‐Score and its five domains in the four subgroups: pwCF aged <12 years with and without ETI (n=10 and n=21, respectively), and pwCF aged ≥12 years with and without ETI (n=23 and n=10, respectively).
