# Supplementary material for: The validated French CFAbd‐Score reveals a lower burden of gastrointestinal symptoms in patients on Elexacaftor/Tezacaftor/Ivacaftor
Source: J Pediatr Gastroenterol Nutr. 2025 Nov 3;82(1):24–32. doi: 10.1002/jpn3.70244 (PMC12780474; doi:10.1002/jpn3.70244)
Supplement: Supplementary file 1 — Table S1. CFAbd‐Score and its five domains for ages ≥12 years in the three subgroups: pwCF without ETI, pwCF on ETI and HC. [file JPN3-82-24-s002.docx]

**Table S1.** CFAbd-Score and its five domains for ages ≥12 years in the three subgroups:
pwCF without ETI, pwCF on ETI and HC

|  | **PwCF without ETI**  **(median, IQR)** |  | **PwCF with ETI**  **(median, IQR)** | **Healthy controls**  **(median, IQR)** |
| --- | --- | --- | --- | --- |
|  | **n=10** |  | **n=23** | **n=56** |
| **Total CFAbd-Score (points)** | 17.3 (7, 30.5) |  | 8.2 (4.8, 11.5) | 9.1 (2.9, 17.1) |
| **Pain (points)** | 17.5 (5, 27.5) |  | 5.0 (0, 20) | 5.0 (0, 20) |
| **GERD (points)** | 20.0 (0, 31.7) |  | 0.0 (0, 6.7) | 6.7 (0, 20) |
| **DBM (points)** | 17.1 (11.5, 25.6) |  | 12.5 (7.5, 16.2) | 10.0 (5, 20) |
| **DA (points)** | 4.5 (0, 18) |  | 0.0 (0, 6) | 0.0 (0, 8) |
| **QoL (points)** | 23.8 (0.6, 41.9) |  | 2.5 (0, 12) | 2.5 (0, 20) |
