# Supplementary material for: The validated French CFAbd‐Score reveals a lower burden of gastrointestinal symptoms in patients on Elexacaftor/Tezacaftor/Ivacaftor
Source: J Pediatr Gastroenterol Nutr. 2025 Nov 3;82(1):24–32. doi: 10.1002/jpn3.70244 (PMC12780474; doi:10.1002/jpn3.70244)
Supplement: Supplementary file 2 — Table S2. CFAbd‐Score and its five domains in the four subgroups: pwCF aged <12 years with and without ETI (n=10 and n=21, respectively), and pwCF aged ≥12 years with and without ETI (n=23 and n=10, respectively). [file JPN3-82-24-s001.docx]

**Table S2.** CFAbd-Score and its five domains in the four subgroups: pwCF aged <12 years with and without ETI (n=10 and n=21, respectively), and pwCF aged ≥12 years with and without ETI (n=23 and n=10, respectively).

|  | **Subgroup** | **Median, IQR** | **p-values** |
| --- | --- | --- | --- |
| **Total CFAbd-Score** | ETI & <12 years | 12.8 (7.6, 17.5) | p>0.05 for all comparisons, except p<0.01 for  **ETI & ≥12 years** vs. **no ETI & <12 years** |
|  | ETI & ≥12 years | **8.2** (4.8, 11.5) |  |
|  | no ETI & <12 years | **18.2** (10.6, 28.6) |  |
|  | no ETI ≥12 years | 17.3 (7, 30.5) |  |
| **Pain** | ETI & <12 years | 15 (1.7, 20) | p>0.05 for all comparisons |
|  | ETI & ≥12 years | 5 (0, 20) |  |
|  | no ETI & <12 years | 25 (0, 35) |  |
|  | no ETI & ≥12 years | 17.5 (5, 27.5) |  |
| **GERD** | ETI & <12 years | 0 (0, 6.7) | p>0.05 for all comparisons |
|  | ETI & ≥12 years | 0 (0, 6.7) |  |
|  | no ETI & <12 years | 6.7 (0, 20) |  |
|  | no ETI ≥12 years | 20 (0, 31.7) |  |
| **DBM** | ETI & <12 years | 22.5 (8.9, 31.1) | p>0.05 for all comparisons |
|  | ETI & ≥12 years | 12.5 (7.5, 16.2) |  |
|  | no ETI & <12 years | 22.5 (15, 32.5) |  |
|  | no ETI ≥12 years | 17.1 (11.5, 25.6) |  |
| **DA** | ETI & <12 years | 0 (0, 13) | p>0.05 for all comparisons, except p<0.01 for  **ETI & ≥12 years** vs. **no ETI & <12 years** |
|  | ETI & ≥12 years | **0** (0, 6) |  |
|  | no ETI & <12 years | **12** (4, 20) |  |
|  | no ETI ≥12 years | 4.5 (0, 18) |  |
| **QoL** | ETI & <12 years | 8.8 (0, 15) | p>0.05 for all comparisons |
|  | ETI & ≥12 years | 2.5 (0, 12) |  |
|  | no ETI & <12 years | 8.6 (0, 17.5) |  |
|  | no ETI ≥12 years | 23.8 (0.6, 41.9) |  |
| GERD: Gastroesophageal reflux disease, DBM: Disorders of bowel movement, DA: Disorders of appetite, QoL: Quality of life | | | |
